# A Proteomic Network Approach across the Kidney Stone Disease Reveals Endoplasmic Reticulum Stress and Crystal-Cell Interaction in the Kidney

**DOI:** 10.1155/2019/9307256

**Published:** 2019-10-27

**Authors:** Baoyu Yang, Xiuli Lu, Yang Li, Yuanyuan Li, Daojun Yu, Weiwen Zhang, Chaojie Duan, Kazumi Taguchi, Takahiro Yasui, Kenjiro Kohri, Bing Gao

**Affiliations:** ^1^Department of Biochemistry and Cell Biology, School of Life Sciences, Liaoning University, Shenyang 110036, China; ^2^Key Laboratory of Renal Calcification Disease Prevention and Treatment, Shenyang Medical College, 146 Huanghe North Street, Shenyang 110034, China; ^3^Department of Cell Biology and Genetics, Shenyang Medical College, 146 Huanghe North Street, Shenyang 110034, China; ^4^China-Japan Kidney Stone Research Center, Shenyang Medical College, 146 Huanghe North Street, Shenyang 110034, China; ^5^Department of Nephro-Urology, Nagoya City University Graduate School of Medical Sciences, 1 Kawasumi, Mizuho-ku, Nagoya 467-8601, Japan

## Abstract

Crystal-cell interactions are a vital step toward kidney stone formation. However, its mechanisms remained unclear. Here, a protein-protein interaction (PPI) network analysis of a kidney stone revealed that the proteins were enriched in a posttranslational protein modification process in the endoplasmic reticulum (ER). The *in vitro* study showed that the markers of ER stress, including Bip and CHOP, were upregulated, PERK and ATF6 were activated, and XBP-1 mRNA was spliced. An ER stress-specific protein, caspase-12, was activated in the apoptotic cells induced by calcium oxalate monohydrate (COM) crystals. The treatment with tunicamycin, an ER stress inducer, promoted the crystal-cell adhesion assayed by atomic absorption, reduced cell viability assayed by MTT, and downregulated the expression of proteins involved in the crystal formations. The treatment with salubrinal, an ER stress inhibitor, reversed the above effects for both tunicamycin and COM crystals. The aforementioned main observations were supported by *in vivo* study. These data demonstrated that ER stress was an essentially biological process of crystal-cell interactions. Our findings suggest that blocking ER stress may become a potential approach to preventing a kidney stone.

## 1. Introduction

The incidence of kidney stone disease is about 2–5% of the population in Asia and 8–15% in Europe and North America [1]. Approximately 80% of kidney stone types are calcium oxalate stone. The recurrence rate of a kidney stone may be higher than 50% after five years [2]. The significant challenges for clinicians are to prevent recurrence of kidney stone patients. However, kidney stone formation is a complex response of cells to the exposure to crystals; the precise mechanism causing crystal-cell interactions is still unclear.

Both a network analysis and a gene ontology (GO) analysis are rapidly becoming powerful tools in complex disease studies [3, 4]. It provides a visual framework and protein enrichment for specific functional categories [5]. A large number of proteins are reported to be involved in the process of kidney stone formation. Some macromolecules, such as osteopontin (OPN) [6], matrix Gla protein (MGP) [7], bikunin [8], and Tamm-Horsfall proteins [9], have been identified in both the urine and kidney stone matrix, and their gene variants have also been reported to affect the risk of kidney stone disease [10–12]. Recently, Wright et al. identified more than 1000 proteins by a urinary proteome analysis [13]. These proteins may interact with each other to play a vital role in modulating crystal nucleation, growth, aggregation, and adhesion to renal epithelial cells. It also provides us with a significant amount of unprocessed information for understanding crystal-cell interactions in the process of kidney stone formation.

In the present study, we collected human protein candidates associated with a kidney stone to perform a bioinformatics analysis and found that the candidate proteins were significantly enriched in a posttranslational protein modification process in the ER. The *in vitro* studies showed that markers associated with ER stress, Bip/GRP78 and CHOP, were upregulated, suggesting that ER stress was directly involved in crystal-cell interactions. Caspase-12, an ER stress-specific caspase protein, was activated. Tunicamycin, an ER stress inducer, increased crystal-cell adhesion, reduced cell viability, and downregulated the expression of proteins associated with kidney stone formation. Salubrinal, an ER stress inhibitor, can reverse the above effects of both tunicamycin and COM crystals. The aforementioned main observations were supported by our *in vivo* study. These findings identified an essential mechanism of crystal-cell interaction in kidneys and provided a potential strategy for the prevention and treatment of a kidney stone, by targeting a cellular ER stress pathway.

## 2. Materials and Methods

### 2.1. Protein Dataset

Proteins that are associated with kidney stone formation were obtained from a proteome study and a search in PolySearch using the keywords “kidney stone” or “nephrolithiasis”. The proteome study performed a label-free nano-ultraperformance liquid chromatography between the 57 stone formers and 57 non-stone-forming controls [13]. The protein names were converted into one unified form using “DAVID Bioinformatics Resources”.

### 2.2. PPI Network Construction and GO Analysis

**Figure 1:**
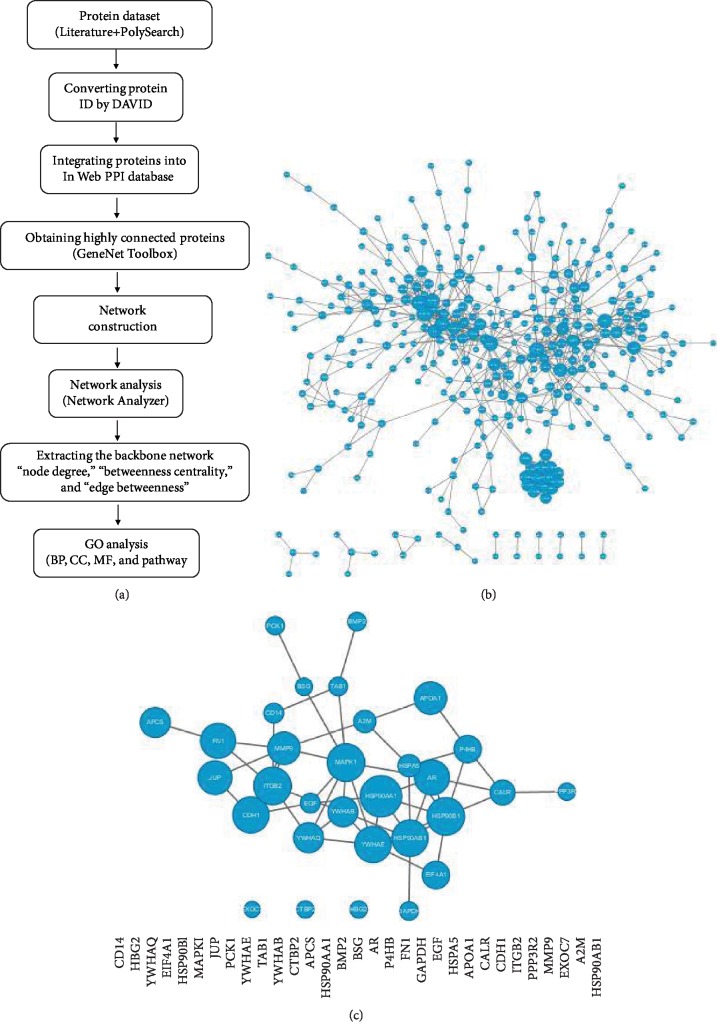
Network analysis of a kidney stone. A flowchart of network construction and a GO analysis (a). The primary kidney stone PPI network was constructed from protein sets obtained from PolySearch and a proteomics data by Wright et al. [13], which consisted of 340 nodes connected via 740 edges (b). The backbone kidney stone PPI network was derived from the primary kidney stone PPI network, which consisted of 31 nodes connected via 51 edges (c). Bigger nodes represent genes with more links.

The candidate proteins were integrated into an “InWeb” PPI database [14]. A permutation test was performed to evaluate whether candidate proteins are significantly linked via PPI networks, rather than by chance. Highly connected proteins were obtained using a GeneNet Toolbox [15]. A backbone network was extracted from the top 10% hub proteins according to rank by the “node degree,” “betweenness centrality,” and “edge betweenness.” The PPI network was visualized using the Cytoscape software [16]. A GO analysis was performed to find protein enrichment using “DAVID Bioinformatics Resources” [17]. GO terms including biological processes (BP), cellular components (CC), and molecular functions (MF) were examined. A flowchart was developed (Figure 1(a)).

### 2.3. Cell Culture

The HK2 (human kidney 2) cells (American Type Culture Collection) were exposed for three, six, 12, 24, and 48 hours to 100 *μ*g/ml calcium oxalate monohydrate (COM) crystals, 1 *μ*g/ml tunicamycin, and 1 *μ*M salubrinal in DMEM, respectively. Tunicamycin, an inhibitor of N-linked glycosylation, can cause cell cycle arrest in a G1 phase to induce an unfolded protein response [18]. Salubrinal is an inhibitor of ER stress, selectively inhibiting eIF2*α* dephosphorylation.

### 2.4. Animal Models

Male Sprague-Dawley (SD) rats were divided into two groups, a treatment group (*n* = 25) and a control group (*n* = 5). The treatment group was administered 0.75% ethylene glycol (EG) in drinking water for up to eight weeks, and the control group was given plain water. Rats were dissected at zero-, two-, four-, six-, and eight-week intervals. The experiment was reviewed and approved by the Shenyang Medical College Animal Committee.

### 2.5. RNA Preparation

The cells were homogenized using a homogenizer (Vibra Cell, Sonics, USA), and total RNA was extracted with an RNAiso kit (Takara, Japan). Total RNA was treated with DNase I for 30 minutes at 37°C, to avoid genomic DNA contamination.

### 2.6. Semiquantitative PCR

A semiquantitative PCR assay was performed using previously described methods [7]. The first-strand cDNA was synthesized from 2 *μ*g of total RNA using an EasyScript First-Strand cDNA Synthesis SuperMix kit (TransGen Biotech, Beijing). The glyceraldehyde-3-phosphate dehydrogenase gene (GAPDH) was used as an endogenous control. The primers were as follows: human XBP-1 (X-box binding protein 1) sense 5′-TTACGAGAGAAAACTCATGGCC-3′ and human XBP-1 antisense 5′-GGGTCCAAGTTGTCCAGAATGC-3′, rat XBP-1 sense 5′-TTACGAGAGAAAACTCATGGGC-3′ and rat XBP-1 antisense 5′-GGGTCCAACTTGTCCAGAATGC-3′, human GAPDH sense 5′-GTCTCCTCTGACTTCAACAGCG-3′ and human GAPDH antisense 5′-ACCACCCTGTTGCTGTAGCCAA-3′, and rat GAPDH sense 5′-TGCTGAGTATGTCGTGGAGTCTA-3′, rat GAPDH antisense 5′-AGTGGGAGTTGCTGTTGAAATC-3′. The assay was performed in triplicate.

### 2.7. Western Blot Analysis

Proteins were isolated from the culture cells by homogenization in RIPA buffer (Solarbio) with protease inhibitors. Primary antibodies—anti-caspase-12 (AB_2069200), anti-caspase-3 (AB_331439), anti-phospho-PERK (PKR-like endoplasmic reticulum kinase, AB_2095853), anti-phospho-P38 (AB_2139682), and anti-phospho-JNK (c-Jun N-terminal kinase, AB_331338) antibodies—were purchased from Cell Signaling (Beverly, MA). A mouse monoclonal anti-Bip/GRP78 (immunoglobulin heavy-chain binding protein, Grp78, AB_398292) antibody was purchased from BD Biosciences (Bedford, MA). Rabbit anti-CHOP (C/EBP homologous protein, AB_1122157) and mouse anti-OPN (AB_2194995) were purchased from Santa Cruz Biotechnology (CA, USA), rabbit polyclonal anti-MGP (cat no. TA339184) was purchased from OriGene, and mouse monoclonal anti-ATF6 (activating transcription factor, AB_2058774) was purchased from Novus. Beta-actin antibodies were used to confirm equal protein loading among samples. All assays were repeated in triplicate.

### 2.8. TUNEL Assay

Apoptotic cells were detected using a terminal deoxynucleotidyl transferase-mediated X-dUTP nick end labeling (TUNEL) method, using the *in situ* apoptosis detection kit (Takara Biomedicals), following the manufacturer's instructions. The cells were observed using a confocal laser-scanning microscope (Leica, Heidelberg, Germany). Evident nuclear staining represented the apoptotic cells.

### 2.9. Measurement of Cell Viability

Cell viability was measured using the MTT assay. Briefly, 1 × 10^6^ cells were plated into a 96-well plate in 100 *μ*l volume, and then, an appropriate concentration of COM crystals, tunicamycin, and salubrinal was added to HK2 cells for two days of exposure, respectively. A labeling dye was added four hours before the endpoint. A stop solution was added to each well to stop the reaction and solubilize the cells. The absorbance was read at 490 nm.

### 2.10. Atomic Absorption Assay

Atomic absorption was performed to assay the effect of COM crystal adhesion to cells. COM crystals were treated with ultrasound for 15 minutes and suspended in DMEM at a final concentration of 2 mg/ml. The cells were exposed to 100 *μ*g/ml COM crystals for five minutes. The nonadherent crystals were removed by rapidly washing the plate three times with the PBS solution. The COM crystals that had adhered to cells were lysed with 5 ml of 6 M HCl. A quantitative analysis of the COM crystals was conducted by measuring the calcium concentration of the supernatants using an atomic absorption analysis.

### 2.11. Statistical Analysis

Candidate proteins were collected from a PolySearch search with *Z* score ≥0.1. A permutation test was performed to evaluate network reliability using the GeneNet Toolbox software. A *P* value of <0.05 and a false discovery rate (FDR) of <10 were considered statistically significant for GO analysis. Comparisons were analyzed using a two-way analysis of variance (ANOVA) with Dunnett's multiple comparison test. A *P* value of ≤0.05 was considered to be significant.

## 3. Results

### 3.1. Construction of the PPI Network of a Kidney Stone

Seven hundred seventy-five proteins were obtained from the proteome study, and 112 human proteins were found through a search conducted in PolySearch. A total of 839 candidate proteins were obtained after a name conversion and removing duplicates, and they were then integrated into the InWeb database to construct a kidney stone PPI network. The network contained 340 nodes and 740 edges, which developed a permutation test at *P* < 0.001 (*n* = 1000 permutations) (Figure 1(b)). Thirty-one hub proteins were extracted to obtain a backbone PPI network of a kidney stone, which presented an underlying biological network of kidney stone formation (Figure 1(c)).

### 3.2. Identification of Biological Processes of Crystal-Cell Interaction

A GO analysis was performed for the thirty-one hub proteins. The GO_BP analysis demonstrated that the proteins had enriched eight biological processes (Table 1). Five of the processes were found to be associated with ER, such as “protein folding” (*P* = 1.33*E* − 05), “protein stabilization” (*P* = 8.13*E* − 05), “ATF6-mediated unfolded protein response” (*P* = 1.03*E* − 04), and “protein folding in endoplasmic reticulum” (*P* = 2.22*E* − 04). The GO_CC analysis showed that the proteins were enriched in the “ER chaperone complex” (*P* = 1.34*E* − 04) and “ER lumen” (*P* = 2.32*E* − 04). The GO_MF analysis found that the proteins had enriched the “unfolded protein binding” (*P* = 1.32*E* − 06). An analysis of the KEGG pathway demonstrated that the proteins were enriched during “protein processing in ER” (*P* = 4.30*E* − 04). These results suggest that a critical biological event in the ER might occur during the process of kidney stone formation.

### 3.3. COM Crystals Activated ER Stress through Three Response Signaling Pathways

**Figure 2:**
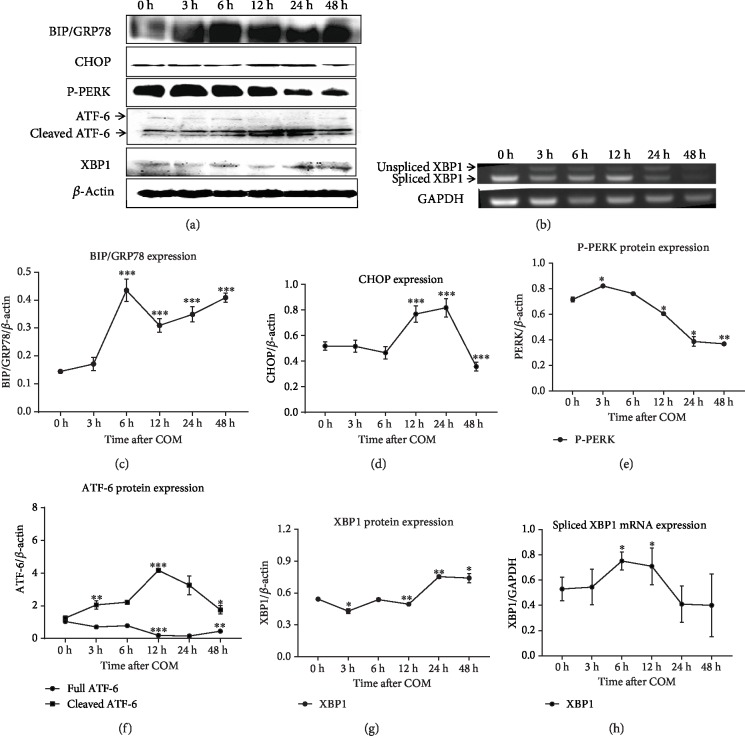
Markers and key sensors of ER stress were activated in HK2 cells under COM crystal exposure. The Bip/GRP78 significantly increased from three to 48 hours (a, c), and the CHOP was increased from 12 to 24 hours, after exposure to COM crystals (a, d). Phospho-PERK (P-PERK) was increased to three hours (a, e). Cleaved ATF6 was increased from three to 48 hours (a, f). XBP-1 was decreased to 12 hours and then increased to 24 hours (a, g). The spliced XBP-1 mRNA was upregulated from three hours to 12 hours (b, h). ^∗^*P* < 0.05, ^∗∗^*P* < 0.01, and ^∗∗∗^*P* < 0.001.

To confirm whether COM crystals activated ER stress, HK2 cells were exposed to crystals for different times and a western blot analysis was performed. The Bip/GRP78, an ER chaperone protein which interacts with initial molecules of unfolded protein responses [19], was meaningfully increased from three to 48 hours, and the peak value presented at six hours (Figures 2(a) and 2(c)). The CHOP, a downstream protein that targets ER stress [19], was significantly upregulated from 12 to 24 hours (Figures 2(a) and 2(d)). These results suggest that ER stress was initiated by exposure to COM crystals.

To clarify which signaling pathway is involved in ER stress induced by COM crystals, we performed a western blot and semiquantitative RT-PCR. Three major ER stress response pathways were investigated, including PERK, inositol-requiring enzyme 1 (IRE1), and ATF6 pathways. PERK is a kinase that phosphorylates eIF2a, leading to a rapid reduction of translational initiation and repression of global protein synthesis [20]. XBP-1 is a transcription factor, and its mRNA is spliced by activated IRE1 resulting in the regulation of expression of some downstream genes [21]. ATF6 is an ER stress-regulated transmembrane transcription factor that is cleaved under ER stress, and the cleaved fragment subsequently moves to the nucleus and binds to DNA as a transcription factor, which causes the transcription of ER chaperones. The PERK protein was strongly activated as early as three hours after COM crystal exposure, and it was gradually decreased from 12 to 48 hours (Figures 2(a) and 2(e)). The full ATF6 protein presented a downward trend from three to 48 hours, whereas the cleaved ATF6 protein increased from three hours and peaked at 12 hours (Figures 2(a) and 2(f)). The level of total XBP-1 protein expression was decreased, and it was then upregulated to 24 hours (Figures 2(a) and 2(g)). The semiquantitative PCR analysis showed that the spliced XBP-1 mRNA was upregulated from three hours to 12 hours (Figures 2(b) and 2(h)). These results demonstrate that COM crystals induce ER stress through at least three major pathways, including PERK, IRE1, and ATF6.

### 3.4. ER Stress-Specific Caspase Was Activated in Apoptotic Cells Induced by COM Crystals

**Figure 3:**
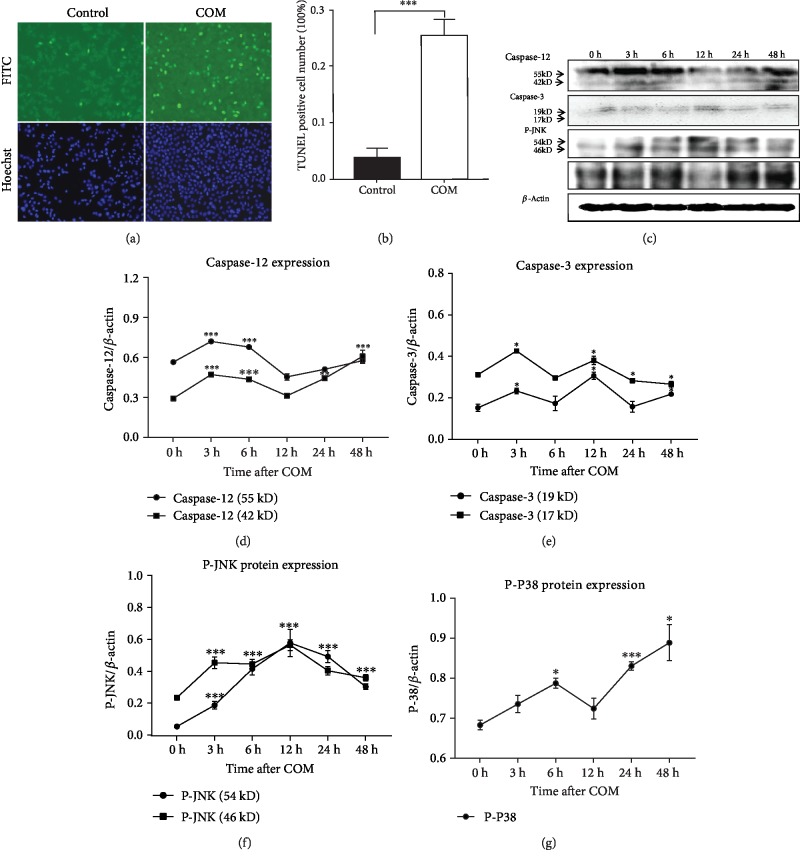
The ER stress-specific caspase was involved in apoptosis induced by COM crystals. Apoptotic cells were detected by the TUNEL and Hoechst assay (a). COM crystal exposure significantly increased apoptotic cell numbers (^∗∗∗^*P* < 0.001) compared with the control group (b). The ER stress-specific caspase-12, caspase-3, phospho-JNK (P-JNK), and phospho-P38 (P-P38) expression was all detected in a time-dependent manner using a western blot (c–g). ^∗∗∗^*P* < 0.001, ^∗^*P* < 0.05.

To understand whether the ER-related proteins were involved in crystal-induced apoptosis, HK2 cells were exposed to COM crystals for 24 hours, and apoptosis was detected by a TUNEL and Hoechst stain assay (Figure 3(a)). The number of apoptotic cells represented by the strong fluorescent signals in the COM-treated group was significantly more than what was found in the control group (Figure 3(b)). Activated caspase-12, an ER stress-specific caspase that mediates ER stress-induced apoptosis, increased markedly after three hours (Figures 3(c) and 3(d)). The cleaved caspase-3, a converging point of an apoptotic pathway, was increased at three and 12 hours, respectively (Figures 3(c) and 3(e)). The JNK and P38, stress-related proteins, significantly increased compared with the control group (Figures 3(c), 3(f), and 3(g)). These results suggest that ER stress was associated with COM crystal-induced apoptosis.

### 3.5. The Effect of ER Stress on Crystal-Cell Interaction

**Figure 4:**
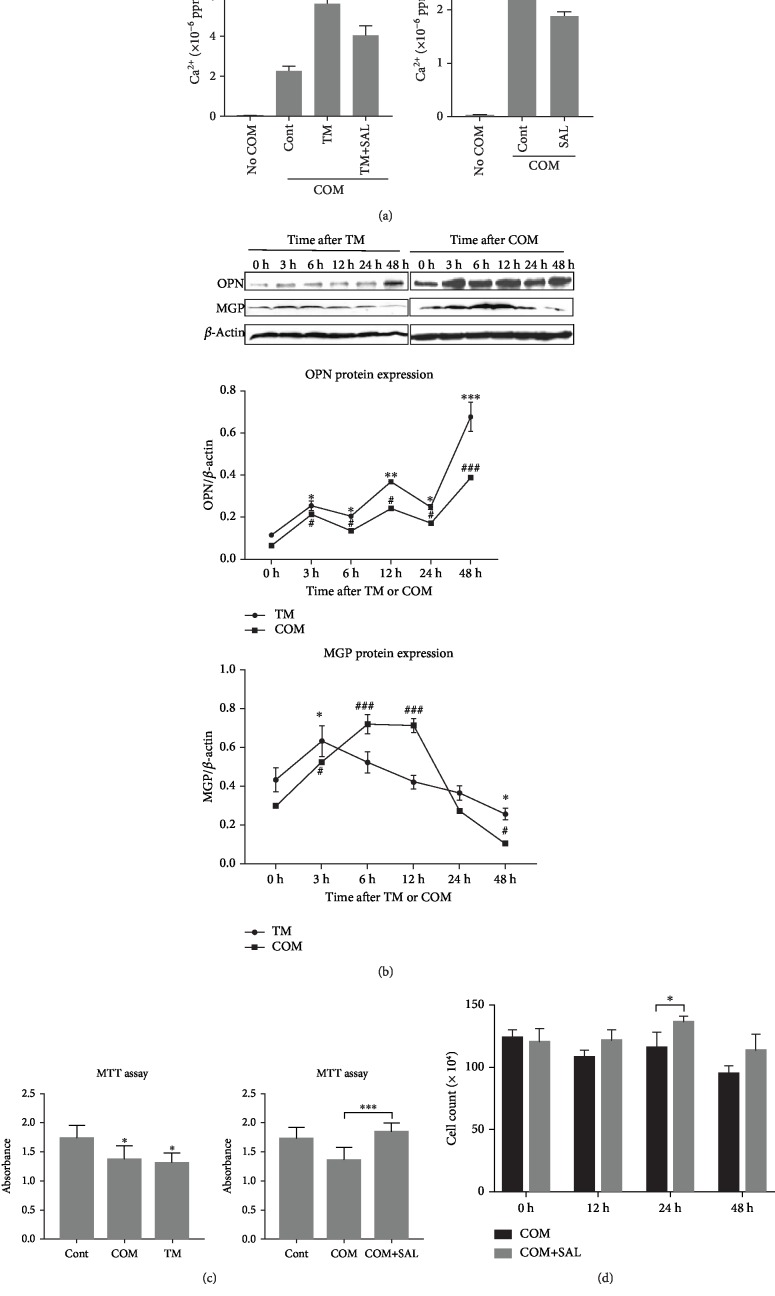
The effect of ER stress on crystal-cell interaction. (a) ER stress promoted crystal-cell adhesion. In the TM+COM group, the HK2 cells were treated with COM crystals for five minutes after exposing TM, an inducer of ER stress, for 24 hours. The COM crystals that adhered to the cells were lysed with 5 ml of 6 M HCl. A quantitative analysis of the COM crystals was conducted, by measuring the concentration using an atomic absorption method. Crystal adhesion in the TM+COM group rose significantly compared with the COM group (^∗∗∗^*P* < 0.001), and SAL reversed it (^∗∗^*P* < 0.01). SAL also reduced crystal-cell adhesion after exposure to COM crystals (^∗^*P* < 0.05). (b) ER stress induced by TM affected the expression of proteins associated with the formation of a kidney stone. Protein expression showed a similar trend between TM exposure and COM exposure. ^∗,#^*P* < 0.05, ^∗∗,##^*P* < 0.01, and ^∗∗∗,###^*P* < 0.001 vs. 0 h. (c) ER stress induced by TM reduced cell viability, and SAL reversed it *in vitro*. HK2 cells were exposed to 100 *μ*g/ml COM crystals and 1 *μ*g/ml TM for two days, respectively. COM crystals and TM significantly reduced cell viability (^∗^*P* < 0.05). HK2 cells were exposed to 100 *μ*g/ml COM crystals and 1 *μ*M SAL+100 *μ*g/ml COM crystals for two days, respectively. SAL significantly increased cell viability compared with the group of COM crystals (^∗∗∗^*P* < 0.001). (d) The number of cells was compared to that of the group of COM and the group of COM+SAL for 12, 24, and 48 hours. The number of cells in the COM+SAL group significantly increased at 24 hours (^∗^*P* < 0.05). TM represented tunicamycin. SAL represented salubrinal.

To understand the effect of ER stress on crystal-cell adhesion, we analyzed the calcium concentration of crystals that adhered to HK2 cells treated with tunicamycin using an atomic absorption method. Crystal-cell adhesion is a crucial step for kidney stone formation. Quantitative crystal analysis showed that the calcium content remaining on the cell surface of the tunicamycin group significantly increased, compared with that of the control group cells (Figure 4(a)). The treatment with salubrinal markedly reversed the effects of ER stress on crystal-cell adhesion (Figure 4(a)).

To investigate the effect of ER stress on the secretory proteins involved in crystal formation, we treated the HK2 cells with tunicamycin for three, six, 12, 24, and 48 hours and detected the expressions of OPN and MGP. OPN is the main stone matrix component, and MGP is a determined protein of extracellular matrix calcification. OPN expression picked up slowly, and MGP expression increased temporarily during the first three hours, and then, it gradually decreased after exposure to tunicamycin (Figure 4(b)). The pattern of expression in either OPN or MGP was similar to the treatment with COM crystals and the treatment with tunicamycin (Figure 4(b)).

To understand the effect of ER stress on cell viability, the MTT assay was performed.  Figure 4(c) shows that the treatment with both COM crystals and tunicamycin reduced absorbance compared to the control cells (*P* < 0.05). Similarly, treatment with salubrinal meaningfully reversed the effects of COM crystals on cell viability (Figure 4(c)). Also, salubrinal significantly increased the number of viable cells, compared to the group that was only exposed to COM crystals, in the 24th hour (Figure 4(d)). These results suggest that ER stress induced by both tunicamycin and calcium oxalate may promote crystal formation by increasing crystal-cell adhesion, affecting the expression of proteins involved in crystal formation such as OPN and MGP and reducing cell viability. The inhibition of ER stress can, therefore, reduce crystal formation.

### 3.6. ER Stress Occurs in Rats with Crystal Formation

**Figure 5:**
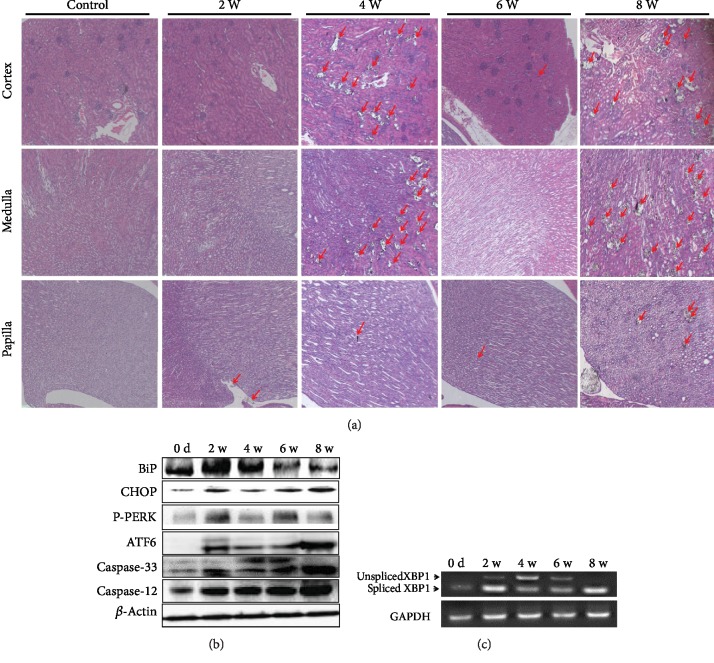
Marker proteins of ER stress expressed changes in rats treated with 0.75% EG from day zero to eight weeks. Crystals were formed from the 2nd week. The red arrow points to the crystal (a). The Bip/GRP78, CHOP, phospho-PERK (P-PERK), ATF6, caspase-3, and caspase-12 were activated (b). XBP-1 was spliced (c).

To confirm the above findings *in vivo*, SD rats were treated with 0.75% EG for two, four, six, and eight weeks. The formation of crystals was observed from the second week (Figure 5(a)). Similar to that found *in vitro*, the Bip/GRP78 and CHOP were increased from the second to the 8th week. The PERK and ATF6 were strongly activated from the second week, and caspase-12 and caspase-3 were cleaved from the second to the eighth week (Figure 5(b)). XBP-1 was spliced at the second to the 8th week (Figure 5(c)). These results demonstrate that ER stress was also induced in rats with crystal formation, through all of the three central ER stress pathways.

## 4. Discussion

In the present study, we performed a kidney stone network analysis and a GO analysis. The study found that the candidate proteins were enriched in a posttranslational protein modification process in the ER. Our *in vitro* and *in vivo* study found that the markers of ER stress, Bip/GRP78 and CHOP, increased the expression in response to COM exposure, which is accompanied by the activation of all of the three major ER stress signaling pathways, including PERK, XBP-1, and ATF6. An ER stress-specific caspase protein was activated in apoptotic cells induced by COM crystals. We further investigated the effect of ER stress on crystal-cell interaction and found that ER stress led to reducing cell viability and affecting the expression of secretory proteins associated with crystal formation and increasing crystal-cell adhesion. These findings suggest that ER stress contributes to crystal formation. Furthermore, an inhibitor of ER stress can reverse cell viability and reduce crystal-cell adhesion. These findings suggest that inhibitors to ER stress may become a promising strategy for the prevention and treatment of a kidney stone.

Omics studies of a kidney stone have presented vast superiority in higher throughput and larger scale compared with traditional studies. Moreover, it may provide us with a panorama for interaction between the proteins and clues for finding a novel mechanism of the disease. However, network analysis may also provide us with false information due to candidate proteins linked via PPIs by chance. In the present study, we performed a permutation test to evaluate whether candidate proteins are significantly linked via PPIs, rather than discovering an outcome by chance. This approach provided us with a network that was as realistic as possible. We also extracted the backbone network from the primary kidney stone network to obtain more essential proteins associated with a kidney stone. Hence, these strategies have significantly improved the reliability of the subsequent GO analysis.

ER stress has been found in various diseases such as diabetes, inflammation, and neurodegenerative disorders. However, concerning a kidney stone, there is little evidence. Hyperoxaluria is a major metabolic promoter of kidney stone formation. A previous study found that the oxalate toxicity increased GRP78 and CHOP expression at an mRNA level in NRK49F cells [22]. High oxalate concentrations are toxic to renal tubular cells, resulting in necrotic cell death and increasing the risk of stone formation [23]. The current study clarified, for the first time, that ER stress is directly involved in crystal-cell interaction, and the apoptotic pathway in renal tubular cells induced by COM crystals. These findings suggest that ER stress occurs in the full process of kidney stone formation.

It is believed that the apoptosis of renal tubular epithelial cells might facilitate the formation of the nucleus of the crystal due to the attachment of calcium ions to the phosphatidylserine, which flips out of the cell membrane during the early stages of apoptosis [24]. However, the precise mechanism regarding the apoptosis of renal tubular epithelial cells induced by COM remains unknown. Our results demonstrated that ER stress induced by COM crystals was associated with caspase-12 activation. Following its activation at the ER, caspase-12 directly cleaves procaspase-9, leading to caspase-9-dependent activation of caspase-3. Also, ER stress-induced apoptosis is regulated by both the CHOP and the ATF6 pathway. CHOP, a downstream of the PERK pathway, is a transcription factor that inhibits the expression of Bcl-2 and promotes apoptosis [25]. CHOP has been found to induce the expression of numerous proapoptotic factors, which promotes protein synthesis and oxidative stress [26]. To confirm the effect of COM crystals on stress-related cell signaling, we investigated the activation of JNK. The activation of JNK significantly increased, compared with the control cells. Activated JNK induces the expression of inflammatory genes by phosphorylating the transcription factor activator protein to promote apoptosis [19, 27]. Constant ER stress can lead to further cell apoptosis, inflammation, and oxidant production.

Increasing evidence shows that some proteins may play critical modulating roles in the development of a kidney stone [7, 28]. OPN is a natural inhibitor of abnormal calcification, and it plays a vital inhibitory role in the phases of stone formation, including crystallization, crystal retention, crystal congregation, and stone formation *in vitro* or *in vivo* [28]. MGP is a molecular determinant regulating calcification of the extracellular matrix. Crystals were observed to form in the renal tubules with a lack of MGP expression, whereas crystals barely formed in the renal tubules with regular MGP expression [7]. In the current study, we analyzed the expressions of these two proteins in ER stress cells induced by tunicamycin. Our results showed that ER stress induced by tunicamycin finally affected these proteins' expression and promoted crystal formation. We believe that both TM and COM could initially upregulate the expression of MGP and OPN to avoid crystal formation, and continuous exposure to TM or COM may induce feedback that increases the ER stress thus inducing apoptosis and reducing the capability of the cell to manage COM.

## 5. Conclusions

The crystal-cell interactions are a crucial step toward kidney stone formation. This study seeks to explore what biological processes contribute to crystal-cell interaction. We find that ER stress may increase the risk of crystal formation by being involved in cell apoptosis, attenuating the protein expression associated with kidney stone formation, reducing cell viability, and increasing crystal-cell adhesion. Our findings provide a potential strategy for the prevention and treatment of kidney stones by targeting cellular ER stress pathways.

## Figures and Tables

**Table 1:** Gene Ontology analysis for the backbone network of a kidney stone.

Term	Count	%	*P* value	Benjamini	FDR	Genes
GOTERM_BP_DIRECT						
GO:0022617~extracellular matrix disassembly	5	16.129	8.45*E* − 06	0.00493	0.012443	BSG, A2M, MMP9, CDH1, FN1
GO:0006457~protein folding	6	19.3548	1.33*E* − 05	0.00388	0.019533	HSP90AB1, P4HB, HSP90B1, HSP90AA1, APCS, CALR
GO:0050821~protein stabilization	5	16.129	8.13*E* − 05	0.01573	0.119559	HSP90AB1, HSP90AA1, APOA1, CALR, GAPDH
GO:0036500~ATF6-mediated unfolded protein response	3	9.67742	1.03*E* − 04	0.01495	0.151433	HSP90B1, HSPA5, CALR
GO:0006950~response to stress	4	12.9032	1.64*E* − 04	0.01897	0.240659	HSP90AB1, MAPK1, HSP90B1, HSP90AA1
GO:0006898~receptor-mediated endocytosis	5	16.129	1.85*E* − 04	0.01786	0.271641	HSP90B1, HSP90AA1, APOA1, CALR, CD14
GO:0034975~protein folding in endoplasmic reticulum	3	9.67742	2.22*E* − 04	0.01839	0.326434	HSP90B1, HSPA5, CALR
GO:1900034~regulation of cellular response to heat	4	12.9032	2.88*E* − 04	0.02084	0.422888	HSP90AB1, MAPK1, HSP90AA1, YWHAE
GOTERM_CC_DIRECT						
GO:0070062~extracellular exosome	24	77.4194	3.39*E* − 14	4.40*E* − 12	3.94*E* − 11	HSP90AB1, P4HB, A2M, BSG, APCS, HSP90AA1, MMP9, YWHAB, CDH1, ITGB2, CALR, YWHAE, PCK1, JUP, MAPK1, HSP90B1, APOA1, EIF4A1, YWHAQ, HSPA5, EGF, GAPDH, CD14, FN1
GO:0042470~melanosome	8	25.8065	1.85*E* − 10	1.20*E* − 08	2.16*E* − 07	HSP90AB1, P4HB, HSP90B1, BSG, HSP90AA1, YWHAB, HSPA5, YWHAE
GO:0005925~focal adhesion	11	35.4839	2.87*E* − 10	1.24*E* − 08	3.34*E* − 07	JUP, MAPK1, P4HB, HSP90B1, BSG, YWHAB, YWHAQ, CDH1, HSPA5, CALR, YWHAE
GO:0031012~extracellular matrix	10	32.2581	5.97*E* − 10	1.94*E* − 08	6.95*E* − 07	JUP, P4HB, HSP90B1, HSP90AA1, APCS, EIF4A1, HSPA5, CALR, GAPDH, FN1
GO:0071682~endocytic vesicle lumen	4	12.9032	2.01*E* − 06	5.22*E* − 05	0.002338	HSP90B1, HSP90AA1, APOA1, CALR
GO:0005829~cytosol	18	58.0645	2.02*E* − 06	4.38*E* − 05	0.002354	HSP90AB1, AR, A2M, HSP90AA1, EXOC7, YWHAB, CALR, TAB1, YWHAE, PCK1, JUP, MAPK1, HSP90B1, APOA1, EIF4A1, YWHAQ, HBG2, GAPDH
GO:0005576~extracellular region	13	41.9355	2.69*E* − 06	4.99*E* − 05	0.003128	P4HB, A2M, BMP2, APCS, HSP90AA1, MMP9, CDH1, CALR, HSP90B1, APOA1, EGF, CD14, FN1
GO:0005913~cell-cell adherens junction	7	22.5807	1.00*E* − 05	1.63*E* − 04	0.011684	HSP90AB1, JUP, BSG, YWHAB, CDH1, HSPA5, YWHAE
GO:0016020~membrane	14	45.1613	1.18*E* − 05	1.70*E* − 04	0.013736	HSP90AB1, HSP90B1, BSG, HSP90AA1, EXOC7, EIF4A1, YWHAB, YWHAQ, CDH1, ITGB2, HSPA5, CALR, GAPDH, YWHAE
GO:0072562~blood microparticle	5	16.129	7.86*E* − 05	0.00102	0.091484	A2M, APOA1, APCS, HBG2, FN1
GO:0034663~endoplasmic reticulum chaperone complex	3	9.67742	1.34*E* − 04	0.00158	0.155484	P4HB, HSP90B1, HSPA5
GO:0009986~cell surface	7	22.5807	2.14*E* − 04	0.00232	0.249403	HSP90AB1, BMP2, HSP90AA1, APOA1, ITGB2, HSPA5, CALR
GO:0005788~endoplasmic reticulum lumen	5	16.129	2.32*E* − 04	0.00232	0.269618	P4HB, HSP90B1, APOA1, HSPA5, CALR
GOTERM_MF_DIRECT						
GO:0005515~protein binding	27	87.0968	4.47*E* − 08	8.09*E* − 06	5.51*E* − 05	HSP90AB1, A2M, EXOC7, MMP9, ITGB2, CDH1, CALR, APOA1, HSPA5, EGF, GAPDH, FN1, P4HB, AR, BSG, BMP2, CTBP2, HSP90AA1, YWHAB, TAB1, YWHAE, JUP, MAPK1, HSP90B1, EIF4A1, YWHAQ, CD14
GO:0001948~glycoprotein binding	6	19.3548	9.67*E* − 08	8.75*E* − 06	1.19*E* − 04	HSP90AB1, HSP90AA1, CDH1, ITGB2, HSPA5, CALR
GO:0051082~unfolded protein binding	6	19.3548	1.32*E* − 06	7.96*E* − 05	0.001626	HSP90AB1, HSP90B1, HSP90AA1, APCS, HSPA5, CALR
GO:0098641~cadherin binding involved in cell-cell adhesion	7	22.5807	1.24*E* − 05	5.62*E* − 04	0.015318	HSP90AB1, JUP, BSG, YWHAB, CDH1, HSPA5, YWHAE
GO:0019899~enzyme binding	7	22.5807	2.62*E* − 05	9.50*E* − 04	0.032354	P4HB, AR, A2M, APOA1, YWHAB, HSPA5, YWHAE
GO:0023026~MHC class II protein complex binding	3	9.67742	3.86*E* − 04	0.01158	0.47498	HSP90AB1, HSP90AA1, YWHAE
KEGG_PATHWAY						
hsa05200: pathways in cancer	12	38.7097	1.00*E* − 07	1.36*E* − 05	1.17*E* − 04	HSP90AB1, JUP, MAPK1, AR, HSP90B1, BMP2, HSP90AA1, CTBP2, MMP9, CDH1, EGF, FN1
hsa04151: PI3K-Akt signaling pathway	10	32.2581	3.66*E* − 06	2.49*E* − 04	0.004295	HSP90AB1, MAPK1, HSP90B1, HSP90AA1, YWHAB, YWHAQ, EGF, YWHAE, FN1, PCK1
hsa05215: prostate cancer	6	19.3548	1.93*E* − 05	8.76*E* − 04	0.022701	HSP90AB1, MAPK1, AR, HSP90B1, HSP90AA1, EGF
hsa04114: oocyte meiosis	6	19.3548	5.45*E* − 05	0.00185	0.063994	MAPK1, AR, YWHAB, YWHAQ, PPP3R2, YWHAE
hsa04621: NOD-like receptor signaling pathway	5	16.129	5.50*E* − 05	0.0015	0.064592	HSP90AB1, MAPK1, HSP90B1, HSP90AA1, TAB1
hsa04390: Hippo signaling pathway	6	19.3548	2.55*E* − 04	0.00577	0.299238	BMP2, YWHAB, YWHAQ, CDH1, ITGB2, YWHAE
hsa04141: protein processing in endoplasmic reticulum	6	19.3548	4.30*E* − 04	0.00833	0.503915	HSP90AB1, P4HB, HSP90B1, HSP90AA1, HSPA5, CALR
hsa05219: bladder cancer	4	12.9032	5.14*E* − 04	0.0087	0.601459	MAPK1, MMP9, CDH1, EGF
hsa04915: estrogen signaling pathway	5	16.129	5.40*E* − 04	0.00813	0.632287	HSP90AB1, MAPK1, HSP90B1, HSP90AA1, MMP9

## Data Availability

The data used to support the findings of this study are included within the article.

## Abbreviations

ER: Endoplasmic reticulum
PPI: Protein-protein interaction
MGP: Matrix Gla protein
OPN: Osteopontin
SD: Sprague-Dawley
HE: Hematoxylin/eosin
HK2: Human kidney 2
COM: Calcium oxalate monohydrate
DAPI: 4′,6-diamidino-2-phenylindole
MTT: 3-(4,5-dimethylthiazol-2-yl)-2,5-diphenyltetrazolium bromide
TM: Tunicamycin
SAL: Salubrinal
GO: Gene ontology
TUNEL: Terminal deoxynucleotidyl transferase-mediated X-dUTP nick end labeling
PCR: Polymerase chain reaction
Bip/GRP78: Immunoglobulin heavy-chain binding protein
GAPDH: Glyceraldehyde-3-phosphate dehydrogenase
PERK: PKR-like endoplasmic reticulum kinase
XBP-1: X-box binding protein 1
CHOP: C/EBP homologous protein
ATF6: Activating transcription factor
JNK: c-Jun N-terminal kinase
FDR: False discovery rate.
